# Improved Visualization of Pancreatic Cystic Lesions on Magnetic Resonance Cholangiopancreatography Using Super-Resolution Deep Learning Reconstruction

**DOI:** 10.1007/s10278-025-01708-y

**Published:** 2025-10-09

**Authors:** Jun Kanzawa, Koichiro Yasaka, Yusuke Asari, Mai Sato, Saori Koshino, Yuki Sonoda, Shigeru Kiryu, Osamu Abe

**Affiliations:** 1 Department of Radiology, University of Tokyo Hospital, Tokyo, Japan; 2 Department of Radiology, International University of Health and Welfare, Chiba, Japan

**Keywords:** Pancreatic cystic lesions, Deep learning, Super-resolution deep learning reconstruction, Magnetic resonance imaging, Magnetic resonance cholangiopancreatography

## Abstract

To assess the efficacy of super-resolution deep learning reconstruction (SR-DLR) in enhancing the visualization of pancreatic cystic lesions (PCLs) on magnetic resonance cholangiopancreatography (MRCP). This retrospective study included 85 patients who underwent MRCP, comprising 52 patients with PCLs and 33 without. Images reconstructed using SR-DLR were compared with original images. Quantitative metrics included signal-to-noise ratio (SNR) and contrast-to-noise ratio (CNR) of the common bile duct (CBD) and PCLs, as well as full width at half maximum (FWHM), edge rise distance (ERD), and edge rise slope (ERS) of the CBD and main pancreatic duct (MPD). Qualitative evaluation was conducted by three radiologists, assessing the depiction of PCLs and the MPD, image sharpness, noise, artifacts, overall image quality, and the connection of PCLs and MPD. Quantitative and qualitative metrics were compared using paired *t*-test and the Wilcoxon signed rank test. SR-DLR significantly enhanced SNR and CNR (*p* < 0.001). Image sharpness was also enhanced, as shown by lower ERD and higher ERS in both CBD and MPD, together with reduced FWHM of the MPD (*p* < 0.005). Qualitative assessments indicated improved depiction of PCLs and image sharpness with SR-DLR across all readers (*p* ≤ 0.017). Most readers also reported improved visualization of the MPD and reduced noise, and overall quality. There was no statistically significant difference in determining the connectivity between PCLs and the MPD. SR-DLR significantly enhances image quality in MRCP, improving visualization of PCLs. These findings suggest that SR-DLR can contribute to appropriate management of PCLs.

## Introduction

Pancreatic cystic lesions (PCLs) are prevalent clinical manifestations that frequently include premalignant conditions [1]. In particular, intraductal papillary mucinous neoplasms (IPMNs), comprising approximately 25% of PCLs, are recognized as precursors of pancreatic cancer, underscoring the need for appropriate clinical management [2].

Magnetic resonance imaging (MRI) and magnetic resonance cholangiopancreatography (MRCP) play pivotal roles in the evaluation of PCLs, exhibiting superior soft-tissue and contrast resolution compared to computed tomography [3]. PCLs are frequently detected incidentally on MRCP, with a reported prevalence as high as 44.7% [4]. MRCP also facilitates the identification of malignant features such as pancreatic duct dilatation and solid components [3, 5]. Consequently, enhancing the image quality of MRCP is paramount for optimizing the clinical management of PCLs.

Recent studies have explored the application of deep learning technologies in radiology practices, encompassing diagnosis to image processing [6, 7]. Notably, the potential for improving image quality has garnered extensive research attention [6]. Super-resolution deep learning reconstruction (SR-DLR) is a recently developed technique poised to provide enhanced spatial resolution [8].

SR-DLR has demonstrated promising performance across diverse MRI examinations. For instance, it has exhibited improved interobserver agreement on neuroforaminal stenosis using 1.5 T cervical spine MRI [8]. Enhanced image quality achieved with SR-DLR has been reported in cranial nerve depiction on 3D fast asymmetric spin echo images [9] and time-of-flight magnetic resonance angiography [10]. However, its utility in abdominal imaging, particularly in the assessment of PCLs using MRCP, remains unexplored in detail.

This study aimed to evaluate the visualization of PCLs with MRCP reconstructed by SR-DLR algorithm. We hypothesized that SR-DLR would enhance the depiction of PCLs and enhance overall image quality.

## Materials and Methods

This retrospective study was approved by our Institutional Review Board. The requirement for obtaining written informed consent was waived due to the retrospective design.

### Patients

This study included a total of 101 consecutive patients who underwent MRCP between July and October 2024. Patients were considered positive for PCLs if they had at least one cystic lesion measuring ≥ 5 mm in diameter, as small lesions were regarded as clinically insignificant [11]. To avoid potential bias from patients with multiple lesions, 15 individuals with more than three lesions were excluded. In addition, one patient with pancreatic pseudocysts secondary to pancreatitis was excluded, as this study focused specifically on primary PCLs.

**Fig. 1:**
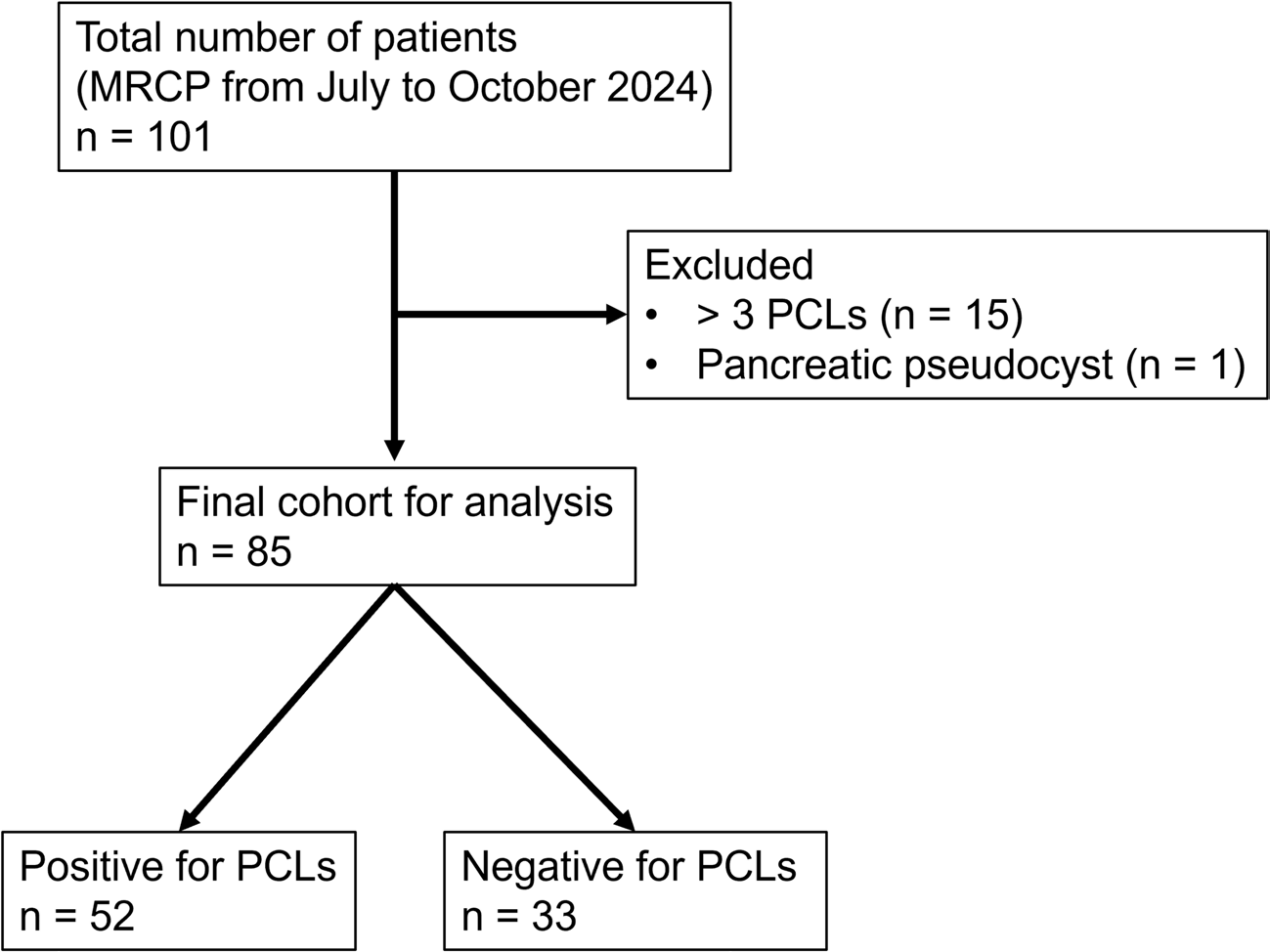
Flow diagram showing the patient selection process. PCL = pancreatic cystic lesion

As a result, a total of 85 patients were included in the final analysis (Fig. 1). Among eligible subjects, 52 were diagnosed as positive for PCLs (28 males and 24 females; mean age: 66.8 ± 11.3 years) and 33 as negative (20 males and 13 females; mean age: 61.2 ± 11.7 years). A total of 68 lesions were included (mean diameter: 13.1 ± 11.5 mm).

Clinical indications for MRCP were as follows: evaluation of PCLs (n = 62), evaluation of pancreatic solid tumors (n = 1), postoperative follow-up of pancreatobiliary maljunction or congenital biliary dilatation (n = 3), assessment of pancreatic or biliary ductal dilatation or stenosis (n = 10), evaluation of gallbladder or hepatic lesions (n = 2), investigation of abdominal pain (n = 1), assessment of elevated pancreatic, hepatic, or biliary enzymes (n = 3), follow-up examination for pancreatitis (n = 3).

### MR Imaging

All patients underwent examination with a 3 T MR imaging system (Vantage Centurian; Canon Medical Systems, Otawara, Japan). MRCP was performed using a 3D acquisition. The scanning parameters were as follows: repetition time, 1,400 ms; echo time, 450 ms; flip angle, 90°; echo train length, 130; acquisition matrix, 320 × 192; pixel bandwidth, 651 Hz; and number of acquisitions, 1. SR-DLR images were reconstructed using Precise IQ Engine (Canon Medical Systems). The reconstruction parameters for SR-DLR were as follows: slice thickness, 2.8 mm; pixel spacing, 0.2604 mm; and row × column, 960 × 1230. Conversely, the reconstruction parameters for the original images were as follows: slice thickness, 2.8 mm; pixel spacing, 0.3906 mm; and row × column, 640 × 820. Both images were reconstructed from the same raw data.

### Overview of the SR-DLR Algorithm

SR-DLR is designed to effectively reduce noise through the utilization of a neural network, followed by fast Fourier transform (FFT), zero-filling of k-space, and inverse FFT. A second neural network is then applied to minimize artifacts that may arise from the zero-filling processes [12]. The datasets used for the training process included images from multiple anatomical regions (such as the brain, lumbar spine, and knee), various imaging contrasts (T1-weighted, T2-weighted, and diffusion-weighted), and different imaging planes (axial, coronal, and sagittal). In total, 45,936 paired MRI datasets were used to train the denoising neural network, and 46,018 paired datasets were used to train the artifact-reduction neural network.

The SR-DLR algorithm was applied as implemented on the MR system, without any additional training or fine-tuning during this study.

### Quantitative Image Analysis

Quantitative image analyses were conducted using ImageJ software (RRID: SCR_003070). A radiologist (with 3 years of post-residency experience in imaging diagnosis) placed regions of interest (ROIs) under the supervision of a senior radiologist (with 14 years of post-residency experience in imaging diagnosis).

Source MRCP images were used for ROI placement. For PCLs, the slice showing the largest diameter was selected. For the CBD and MPD, the slice where the ducts were visualized along their long axis was selected. ROIs for the pancreatic parenchyma were placed on the same slice as the CBD.

**Fig. 2:**
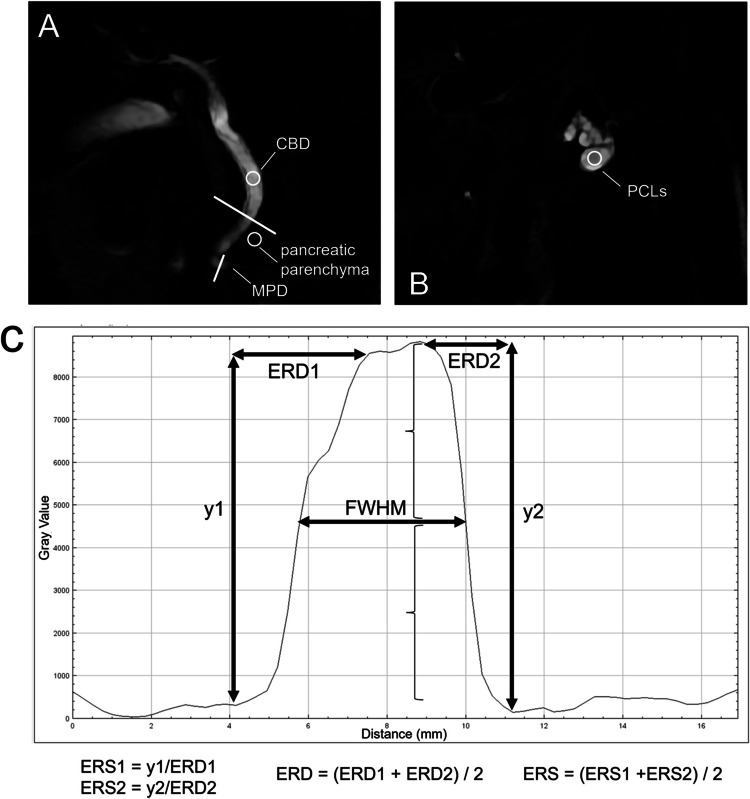
Measurement procedure of quantitative metrics. (**a**) Linear and circular region of interests (ROIs) (white thick line and white circles, respectively) were placed on the common bile duct (CBD). A linear ROI was placed on the distal main pancreatic duct (MPD). A circular ROI was placed on pancreatic parenchyma adjacent to the CBD. (**b**) A circular ROI was placed on pancreatic cystic lesions. (**c**) Signal intensity profiles of linear ROIs were obtained to calculate the edge rise distance (ERD), edge rise slope (ERS), and full width at half maximum (FWHM)

Firstly, circular ROIs of approximately 10-mm diameter were placed on the common bile duct (CBD), PCLs, and pancreatic parenchyma adjacent to the CBD (Fig. 2a and 2b). If the targeted region was smaller than 10 mm in diameter, the largest possible ROI was placed that did not extend to the peripheral parts of the structure. ROIs were placed on SR-DLR images and subsequently copied to the original images. The mean signal intensity (SI) and standard deviation (SD) were recorded.

The signal-to-noise ratio (SNR) and contrast-to-noise ratio (CNR) were calculated as follows (subscripts denote the measured structures) [10]:

$$\begin{array}{l}SNR_{CBD}\:=\:SI_{CBD}/SD_{CBD}\\SN_{RPCL}\:=\:SI_{PCL}/SD_{PCL}\\CNR_{CBD}\:=\:(SI_{CBD}-SI_{PANCREAS})/({(SD_{CBD}\:^2+SD_{PANCREAS}\:^2)/2)}^{1/2}\\CNRPCL\:=\:(SI_{PCL}-SI_{PANCREAS})/({(SD_{PCL}\:^2+\:SD_{PANCREAS}\:^2)/2)}^{1/2}\end{array}$$

Secondly, linear ROIs crossing the distal CBD and MPD were placed on the SR-DLR images (Fig. 2a) and subsequently copied to the original image. The full width at half maximum (FWHM), edge rise distance (ERD), and edge rise slope (ERS) were calculated from the SI profile of linear ROIs (Fig. 2c). ERD and ERS values were averaged for both edges. Superior image sharpness is indicated by lower FWHM, lower ERD, and higher ERS [10, 13].

### Qualitative Image Analysis

Three additional radiologists (readers 1, 2, and 3 with 8, 3, and 1 year of post-residency experience in imaging diagnosis, respectively) participated in qualitative image analysis, who were blinded to the patient background information and the image reconstruction algorithm. The readers evaluated the individual image sets with ImageJ software. In qualitative image analysis, maximum intensity projection images were also included. The image sets were presented in random order. Scores for rating image quality are presented in Table 1. This scoring system was adopted from previous literature investigating deep learning-based reconstruction algorithms [8, 9, 14].

**Table 1:** Scoring system for qualitative image analysis

Evaluation item	Score	Definition
Depiction of PCLs	4	Clear depiction
	3	Slightly blurred
	2	Moderately blurred
	1	Severely blurred
Depiction of MPD	4	Clear depiction
	3	Slightly blurred
	2	Moderately blurred
	1	Severely blurred
Image sharpness	4	Excellent sharpness
	3	Standard sharpness
	2	Reduced sharpness
	1	Poor sharpness
Subjective image noise	4	Minimal noise
	3	Standard noise
	2	More than standard noise
	1	Severe noise
Artifacts	3	Minimal artifact
	2	Standard artifact
	1	Severe artifact
Overall image quality	5	Excellent
	4	Better than standard
	3	Standard
	2	Worse than standard
	1	Poor

*PCL* = pancreatic cystic lesion, *MPD* = main pancreatic duct

### Connectivity of PCLs and Main Pancreatic Duct

A radiologist with 3 years of post-residency experience in imaging diagnosis, together with a senior radiologist with 14 years of experience, established the reference standard for assessing the connectivity between PCLs and the main pancreatic duct (MPD) by consensus.

The same readers who participated in the qualitative image analysis evaluated the connectivity of PCLs and the MPD using the following scale: 4 = connected, 3 = probably connected, 2 = possibly connected, and 1 = not connected.

### Statistical Analysis

Statistical analyses were conducted using EZR version 1.37 (https://www.jichi.ac.jp/saitama-sct/SaitamaHP.files/statmed.html) [15], a graphical user interface of R version 2.4–0 (R Foundation for Statistical Computing, Vienna, Austria RRID: SCR_001905).

Paired *t*-tests were employed to compare quantitative metrics between SR-DLR and the original. The results of qualitative evaluation were compared between SR-DLR and the original using the Wilcoxon signed rank test. A *p*-value less than 0.050 indicated a statistically significant difference for all tests. Interobserver agreement among the three readers for the qualitative assessments was evaluated using Fleiss’ kappa statistics.

The area under the receiver operating characteristic curve (AUC) for determining the connectivity of PCLs and the MPD was compared between SR-DLR and the original images using the DeLong test for each reader.

## Results

### Quantitative Image Analysis

Table 2 presents the results of quantitative image analysis. The SNR and CNR of the CBD and PCLs were both significantly higher in SR-DLR compared to the original images (*p* < 0.001). Regarding linear ROI analysis of the CBD, there was no significant difference in FWHM (*p* = 0.130), whereas ERD was significantly lower and ERS significantly higher in SR-DLR compared to the original images (*p* < 0.001). For the MPD, both FWHM and ERD were significantly lower, and ERS significantly higher, in SR-DLR compared to the original images (*p* < 0.005).

**Table 2:** Results of quantitative image analyses

Reader	Original	SR-DLR	Original *vs.* SR-DLR (*p*)
SNR_CBD_	10.6 ± 4.6	13.4 ± 8.5	< 0.001*
SNR_PCL_	10.3 ± 6.8	12.3 ± 11.7	< 0.001*
CNR_CBD_	8.1 ± 3.4	10.9 ± 5.8	< 0.001*
CNR_PCL_	8.5 ± 5.2	10.4 ± 8.8	< 0.001*
FWHM_CBD_ (mm)	12.3 ± 3.5	12.0 ± 3.6	0.130
ERD_CBD_ (mm)	4.5 ± 1.2	3.5 ± 1.3	< 0.001*
ERS_CBD_ (mm^−1^)	1625.1 ± 710.0	2446.0 ± 1355.7	< 0.001*
FWHM_MPD_ (mm)	2.7 ± 1.0	2.4 ± 0.6	0.005*
ERD_MPD_ (mm)	2.8 ± 0.9	2.74 ± 1.0	0.005*
ERS_MPD_ (mm^−1^)	1527.5 ± 886.2	2192.6 ± 1735.5	< 0.001*

*SNR* = signal-to-noise-ratio, *CNR* = contrast-to-noise ratio. Subscripts denote the anatomical structures measured: CBD indicates the common bile duct, *PCL* the pancreatic cystic lesion, and* MPD* the main pancreatic duct. *SR-DLR* = super-resolution deep learning reconstruction, *FWHM *= full width at half maximum, *ERD* = edge rise distance, *ERS* = edge rise slope. Comparisons between original and *SR-DLR* using paired *t*-tests. *, a statistically significant difference (*p* < 0.05)

### Qualitative Image Analysis

**Fig. 3:**
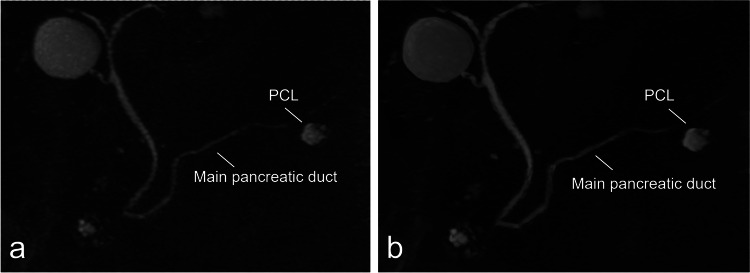
Magnetic resonance cholangiopancreatography of a male patient in his 60 s. (**a**) Maximum intensity projection (MIP) of the original images. (**b**) MIP of the images reconstructed using super-resolution deep learning reconstruction (SR-DLR). The main pancreatic duct and the contour of the pancreatic cystic lesion (PCL) are more clearly demarcated in the SR-DLR image. The depiction of the main pancreatic duct was rated by three readers as 2/2/2 for the original images and 2/4/1 for the SR-DLR images. The depiction of the PCL was rated as 4/3/4 for the original images and 4/4/4 for the SR-DLR images. Higher scores indicate better depiction

**Fig. 4:**
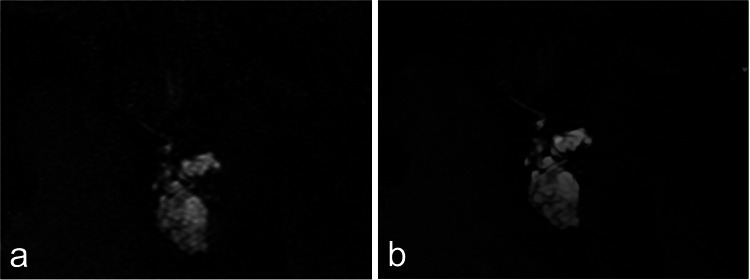
Magnetic resonance cholangiopancreatography of a male patient in his 80 s demonstrating a multilocular pancreatic cystic lesion (PCL). (**a**) Original image. (**b**) Image reconstructed using super-resolution deep learning reconstruction (SR-DLR). Internal septations of the PCL are more clearly delineated in the SR-DLR image. Three readers rated the depiction of the PCL as 4/2/4 for the original images and 4/4/4 for the SR-DLR images. Higher scores indicate better depiction

Table 3 shows the results of qualitative image analyses. The depiction of PCLs and sharpness were scored significantly higher in SR-DLR by all readers (*p* ≤ 0.017). The depiction of MPD, noise, and overall quality were scored significantly higher in SR-DLR by two out of three readers (*p* ≤ 0.009). Artifacts were scored significantly higher in the original images by a reader (*p* < 0.001). Representative cases are presented in Fig. 3 and 4.

**Table 3:** Results of qualitative image analyses

Reader	Original	SR-DLR	Original *vs.* SR-DLR (*p*)	Kappa statistic (95% CI)
Depiction of lesions (score 4/3/2/1)				0.205 (0.120—0.287)
1	44/9/15/0	54/10/4/0	< 0.001*	
2	0/25/42/1	48/18/2/0	< 0.001*	
3	27/21/16/4	36/19/8/5	0.017*	
Depiction of MPD (score 4/3/2/1)				0.263 (0.195–0.328)
1	13/23/40/9	14/30/31/10	0.134	
2	0/27/48/10	45/30/9/1	< 0.001*	
3	16/25/28/16	20/32/17/16	0.006*	
Sharpness (score 4/3/2/1)				0.165 (0.089–0.234)
1	23/43/17/2	50/26/9/0	< 0.001*	
2	0/28/57/0	71/13/1/0	< 0.001*	
3	12/29/39/5	19/37/25/4	0.001*	
Noise (score 4/3/2/1)				0.118 (0.052–0.182)
1	36/33/16/0	38/37/10/0	0.229	
2	0/32/53/0	23/60/2/0	< 0.001*	
3	8/21/51/5	12/41/30/2	< 0.001*	
Artifacts (score 3/2/1)				−0.038 (−0.098 – 0.016)
1	26/46/13	20/53/12	0.360	
2	84/1/0	83/2/0	0.773	
3	40/36/9	22/52/11	< 0.001*	
Overall quality (score 5/4/3/2/1)				0.110 (0.052–0.164)
1	12/11/40/11/11	14/17/39/8/7	0.009*	
2	0/0/32/53/0	25/43/15/2/0	< 0.001*	
3	3/16/35/24/7	6/15/37/20/7	0.208	

Numbers of patients for each score are shown. SR-DLR: super resolution deep learning reconstruction. MPD: main pancreatic duct. CI: confidence interval. Comparisons between original and SR-DLR using Wilcoxon signed-ranks tests. *, a statistically significant difference (*p* < 0.050)

Kappa values ranged from −0.038 to 0.263, indicating slight to fair interobserver agreement across the qualitative assessments.

### Connectivity of PCLs and Main Pancreatic Duct

**Fig. 5:**
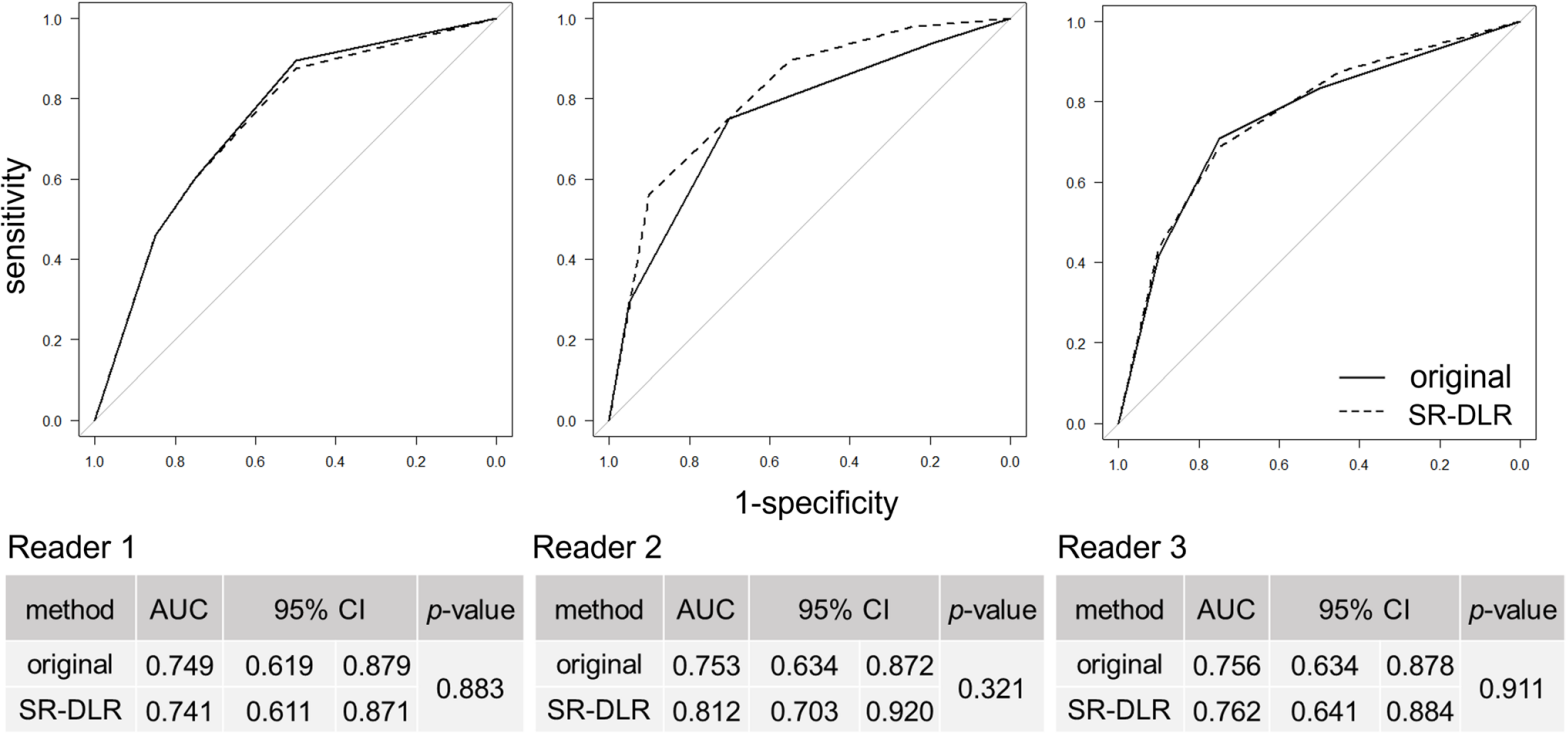
Receiver operating characteristic curves and the corresponding areas under the curve (AUC) for evaluating the connectivity of pancreatic cystic lesion and the main pancreatic duct. Comparisons between original and super-resolution deep learning reconstruction using the DeLong test. CI: confidence interval

Among the PCLs, 48 were connected to the MPD, while 20 were not. Figure 5 presents the receiver operating characteristic (ROC) curves for assessing the connectivity of PCLs and the main pancreatic duct (MPD). For each reader, there was no statistically significant difference in AUC between SR-DLR and original images.

## Discussion

The management of PCLs holds clinically significance due to their high prevalence and potential as precursors of pancreatic cancer. Imaging examination with MRCP plays a pivotal role in both screening and the detailed assessment of malignant features [3]. This study demonstrated that SR-DLR significantly improved image quality in terms of both quantitative metrics and qualitative assessments by radiologists.

This study quantified image sharpness, which is associated with spatial resolution, by measuring ERD, ERS, and FWHM of the CBD and MPD. SR-DLR demonstrated significant improvements in ERD and ERS for both ducts, indicating superior spatial resolution. In contrast, no significant difference was observed in the FWHM of the CBD, whereas the FWHM of the MPD showed significant improvement. This discrepancy may be explained by the relatively large diameter of the CBD, which could have limited the sensitivity of FWHM in detecting subtle structural changes.

Enhanced sharpness was also recognized by radiologists, with all readers reporting a significant improvement. Noise evaluation was performed using the objective metrics of CNR and SNR, both of which showed significant improvement in SR-DLR images. The reduction in noise was also confirmed through subjective assessment by the majority of readers. This simultaneous achievement of noise reduction and spatial resolution enhancement has been demonstrated in previous studies using SR-DLR. For example, Yasaka et al. evaluated cervical spine MRI using SR-DLR, demonstrating improvements in noise metrics such as SNR and CNR. Additionally, they demonstrated enhanced spatial resolution based on both the quantitative metric of ERS and subjective assessments by readers [8]. Similarly, Asari et al. reported that subjective noise and sharpness were both improved in T2*‑weighted images reconstructed using SR-DLR [16].

This study also assessed the depiction of PCLs and the MPD through radiologist evaluation. The majority of readers reported improved visualization of these structures and assigned higher overall image quality scores. Given the malignant potential of PCLs is assessed based on both the morphological features of the lesions and the appearance of the MPD, SR-DLR could facilitate clinical decision-making by enhancing the visibility of anatomical details. On the other hand, no statistically significant difference was observed in assessing the connectivity between PCLs and the MPD. The AUC for the original images already demonstrated moderate performance, suggesting that the potential benefits of SR-DLR may not be apparent for structures that are already well visualized on conventional images.

Assessment of artifacts by radiologists did not show improvement with SR-DLR. This finding indicates that SR-DLR may not fully suppress artifacts, highlighting the importance of appropriate image acquisition to maintain diagnostic quality. In particular, MRCP is highly susceptible to artifacts caused by respiratory motion, which may not have been fully corrected during the image reconstruction process. Interestingly, one reader rated the original images higher for artifact scores. This may be explained by the possibility that the characteristic image texture generated by SR-DLR was interpreted as artifacts; however, further validation is needed to confirm this hypothesis.

Interobserver agreement for qualitative assessments was relatively low across the evaluated metrics. However, this study primarily focused on the improvement of image quality as perceived by individual readers rather than on the consistency of scoring between readers. Therefore, the relatively low interobserver agreement is unlikely to undermine the validity of our findings.

This study had some limitations. Firstly, it was a retrospective study conducted at a single facility, which may limit the generalizability of the results. Secondly, the sample size was relatively small. Evaluation of subtle structures, such as small mural nodules, may benefit from the improved visualization provided by SR-DLR. However, no such cases were available in this study. As MRCP is the primary modality for evaluating PCLs and invasive techniques such as endoscopic ultrasound (EUS) or surgery are performed only in limited situations, MRCP findings themselves were used as the reference standard to assess the connectivity of PCLs and the MPD. Although this approach reflects clinical practice, future studies correlating SR-DLR MRCP findings with EUS or surgical pathology would be of great value. Lastly, the study focused on PCLs, so other pathologies assessed by MRCP were not included. Therefore, our findings require validation in other institutions with larger sample sizes and a broader range of pancreatic pathologies.

In conclusion, SR-DLR enhanced image sharpness, reduced image noise, and improved image quality, resulting in a clearer depiction of structures. Our findings suggest that SR-DLR can contribute to appropriate management of PCLs with MRCP.

## Data Availability

The datasets of this study are available from the corresponding author upon reasonable request.
